# Supplementary material for: Interleukin-18 produced by bone marrow-derived stromal cells supports T-cell acute leukaemia progression
Source: EMBO Mol Med. 2014 Apr 28;6(6):821–34. doi: 10.1002/emmm.201303286 (PMC4203358; doi:10.1002/emmm.201303286)
Supplement: Supplementary file 1 — Supplementary Figure S1 [file emmm0006-0821-sd1.pdf]

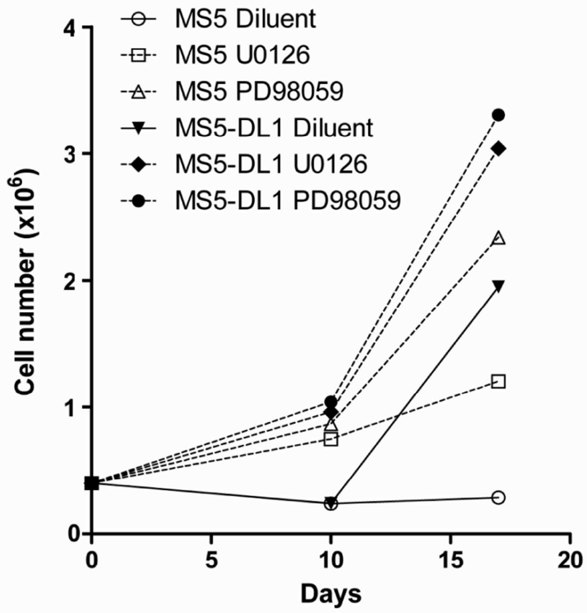


**Figure S1: T-ALL proliferative response to MEK1i**. M18 T-ALL cells ($4x10^5$cells/well) were cultured on MS5 or MS5-DL1 stromal cells in the presence of 20μM U0126 or 10μM PD98059 for two weeks. Cells were counted by FACS analysis at each time point. Representative of two experiments.
