# Supplementary material for: Interleukin-18 produced by bone marrow-derived stromal cells supports T-cell acute leukaemia progression
Source: EMBO Mol Med. 2014 Apr 28;6(6):821–34. doi: 10.1002/emmm.201303286 (PMC4203358; doi:10.1002/emmm.201303286)
Supplement: Supplementary file 2 — Supplementary Figure S2 [file emmm0006-0821-sd2.pdf]

A. OP9

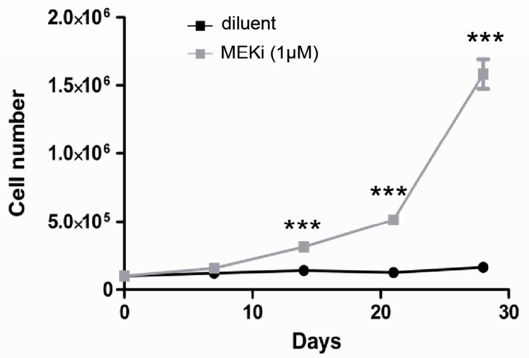

B. Human MSC

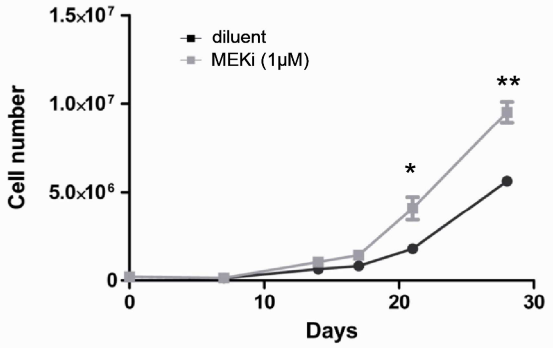

Figure S2: Response of T-ALL cell to MEK inhibition during co-cultures with different support cells. $2x10^5$ T-ALL cells were incubated with OP9 mouse stromal cells (A: M18) or with human mesenchymal stem cells (MSC) (B: M78) in the presence of PD184352 for 28 days. Cells were counted by FACS analysis. *, $p<0.05$; **, $p<0.005$, ***, $p<0.0005$ (Mann and Whitney non-parametric test was used for statistics)
