# Supplementary material for: Interleukin-18 produced by bone marrow-derived stromal cells supports T-cell acute leukaemia progression
Source: EMBO Mol Med. 2014 Apr 28;6(6):821–34. doi: 10.1002/emmm.201303286 (PMC4203358; doi:10.1002/emmm.201303286)
Supplement: Supplementary file 3 — Supplementary Figure S3 [file emmm0006-0821-sd3.pdf]

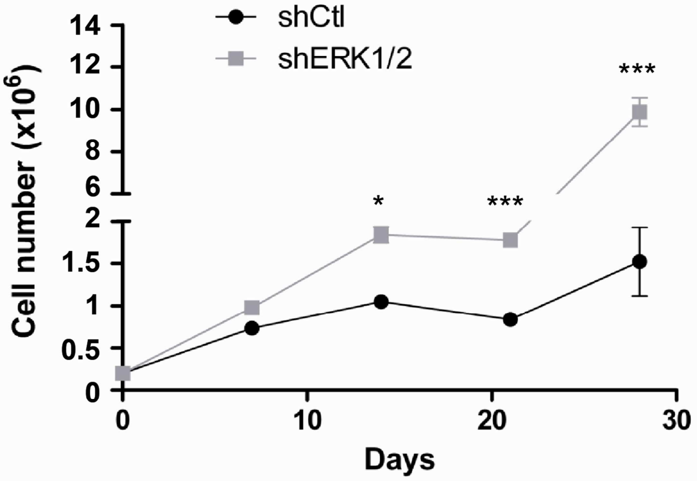

Figure S3: Growth of T-ALL cells cultured on ERK1/2 knock-down MS5 cells. T-ALL M18 cells were cultured on MS5 cells transduced with lentiviral vectors encoding shERK1/2 or shCtl. Cells were counted every week as described in Figure S1. *, $p<0.05$; ***, $p<0.001$ (triplicates) (Mann and Whitney non-parametric test was used for statistics)
