# Supplementary material for: Interleukin-18 produced by bone marrow-derived stromal cells supports T-cell acute leukaemia progression
Source: EMBO Mol Med. 2014 Apr 28;6(6):821–34. doi: 10.1002/emmm.201303286 (PMC4203358; doi:10.1002/emmm.201303286)
Supplement: Supplementary file 4 — Supplementary Figure S4 [file emmm0006-0821-sd4.pdf]

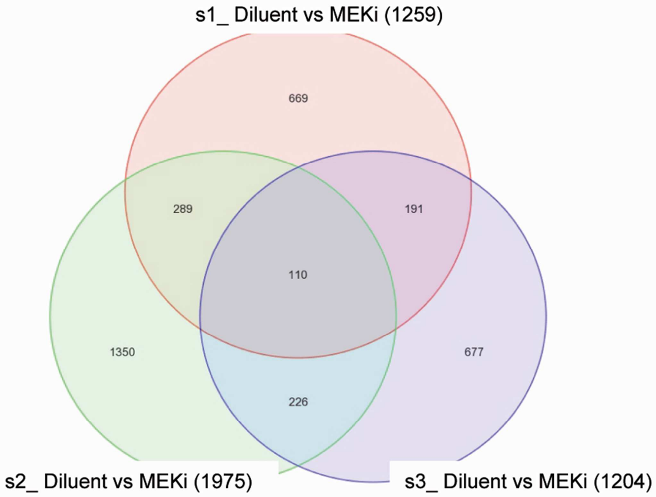

Figure S4: Micro-array analysis of MS5 cells treated or not with MEKi. RNA isolated from MS5 cells cultured in the presence of 1µM PD184352 or DMSO (diluent) for 1 week was hybridized to Affymetrix murine Gene1.0 ST arrays according to the manufacturer's instructions. Three (s1, s2, s3) experiments were performed and processed separately. Results were crossed using pair-wise Tukey's post hoc tests that identified a common set of 110 genes.
