# Supplementary material for: Interleukin-18 produced by bone marrow-derived stromal cells supports T-cell acute leukaemia progression
Source: EMBO Mol Med. 2014 Apr 28;6(6):821–34. doi: 10.1002/emmm.201303286 (PMC4203358; doi:10.1002/emmm.201303286)
Supplement: Supplementary file 5 — Supplementary Figure S5 [file emmm0006-0821-sd5.pdf]

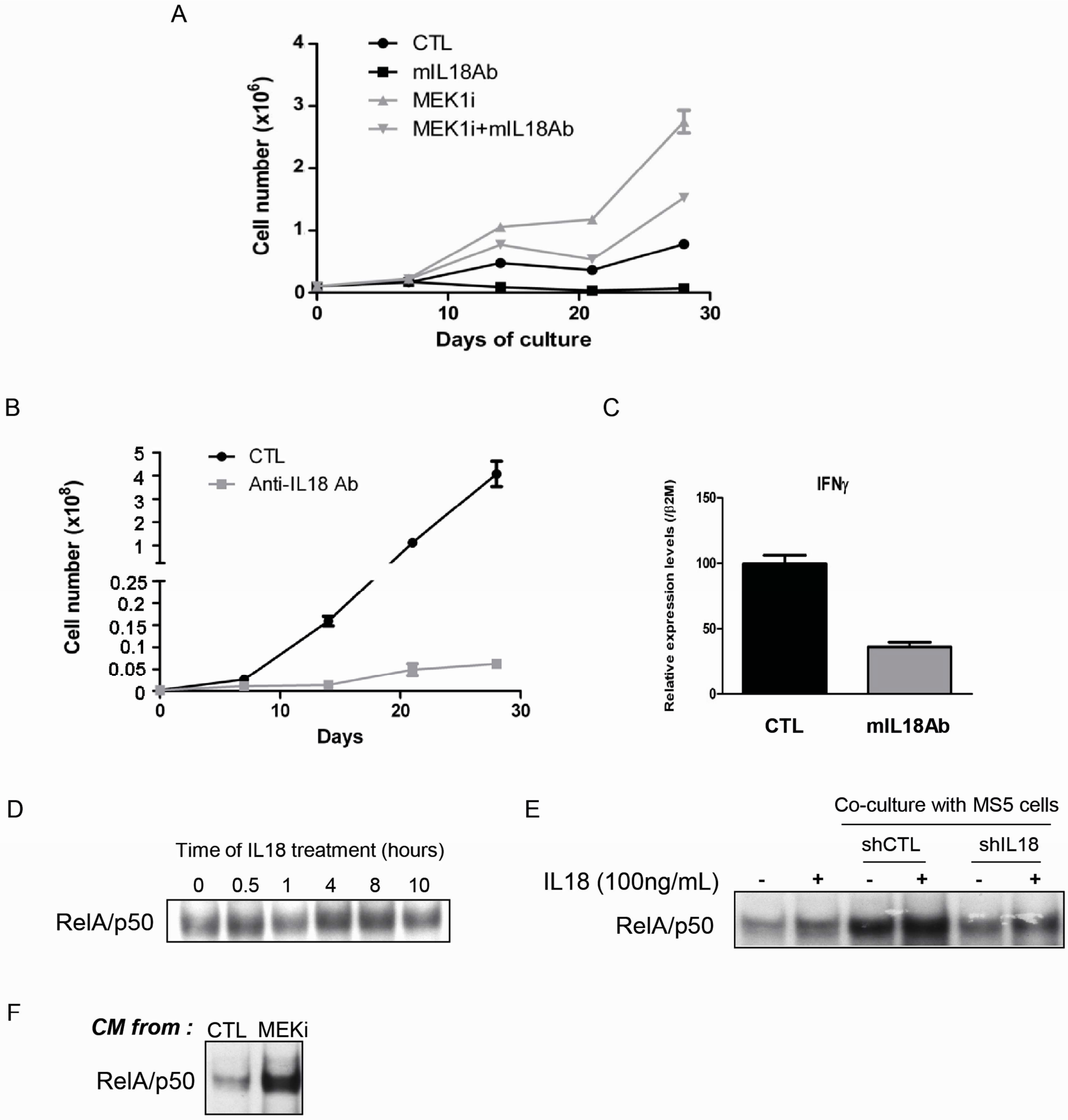

Figure S5: Blocking IL18 decreases T-ALL expansion in vitro. M108 (A) and M105 (B) T-ALL cells were cultured on MS5 cells with or without MEKi (1μM PD184352) and with or without the anti-mouse IL18 antibody (mIL18Ab), as indicated, for 28 days. Cells were counted every week and shown are the cumulative cell numbers. C. IFNγ expression level was measured in M105 T-ALL cells at day 28 of culture. CTL are cells treated with isotype IgG control. D. Kinetic of NF-κB DNA binding activity in M69 T-ALL following incubation with 100ng/mL recombinant human IL18. E. NF-κB activation in M105 T-ALL cells cultured with or without MS5 cells in which IL18 was silenced (shIL18) or not (shCTL) and treated or not with 100ng/mL IL18 for 45 min. F. M105 T-ALL was cultured during 45 minutes with CM from MS5 cells treated every other day during 7 days with or without MEKi.
