# Supplementary material for: Interleukin-18 produced by bone marrow-derived stromal cells supports T-cell acute leukaemia progression
Source: EMBO Mol Med. 2014 Apr 28;6(6):821–34. doi: 10.1002/emmm.201303286 (PMC4203358; doi:10.1002/emmm.201303286)
Supplement: Supplementary file 6 — Supplementary Figure S6 [file emmm0006-0821-sd6.pdf]

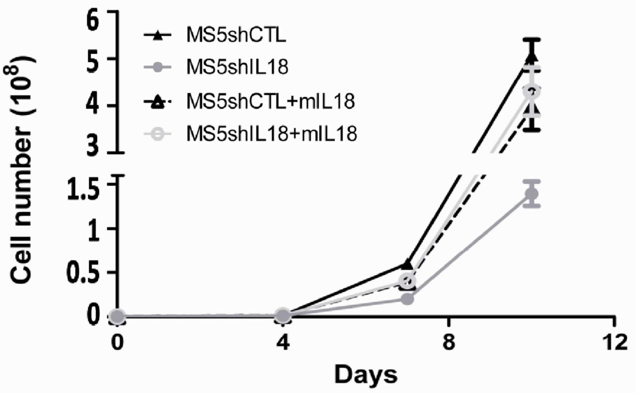

Figure S6: Mouse ICN1+ T-ALL cells are sensitive to IL18. ICN1-induced T-ALL mouse cells were grown on MS5 stromal cells transduced with lentiviral vectors containing a shRNA against IL18 (shIL18) or control (shCTL) and in the presence or not of mouse recombinant IL18. Leukemic cells were harvested at different time points and counted by flow cytometry using anti mouse CD45 antibodies. This result is representative of 2 independent experiments in triplicate. (Mann and Whitney non-parametric test was used for statistics)
