# Supplementary material for: Interleukin-18 produced by bone marrow-derived stromal cells supports T-cell acute leukaemia progression
Source: EMBO Mol Med. 2014 Apr 28;6(6):821–34. doi: 10.1002/emmm.201303286 (PMC4203358; doi:10.1002/emmm.201303286)
Supplement: Supplementary file 7 — Supplementary Figure S7 [file emmm0006-0821-sd7.pdf]

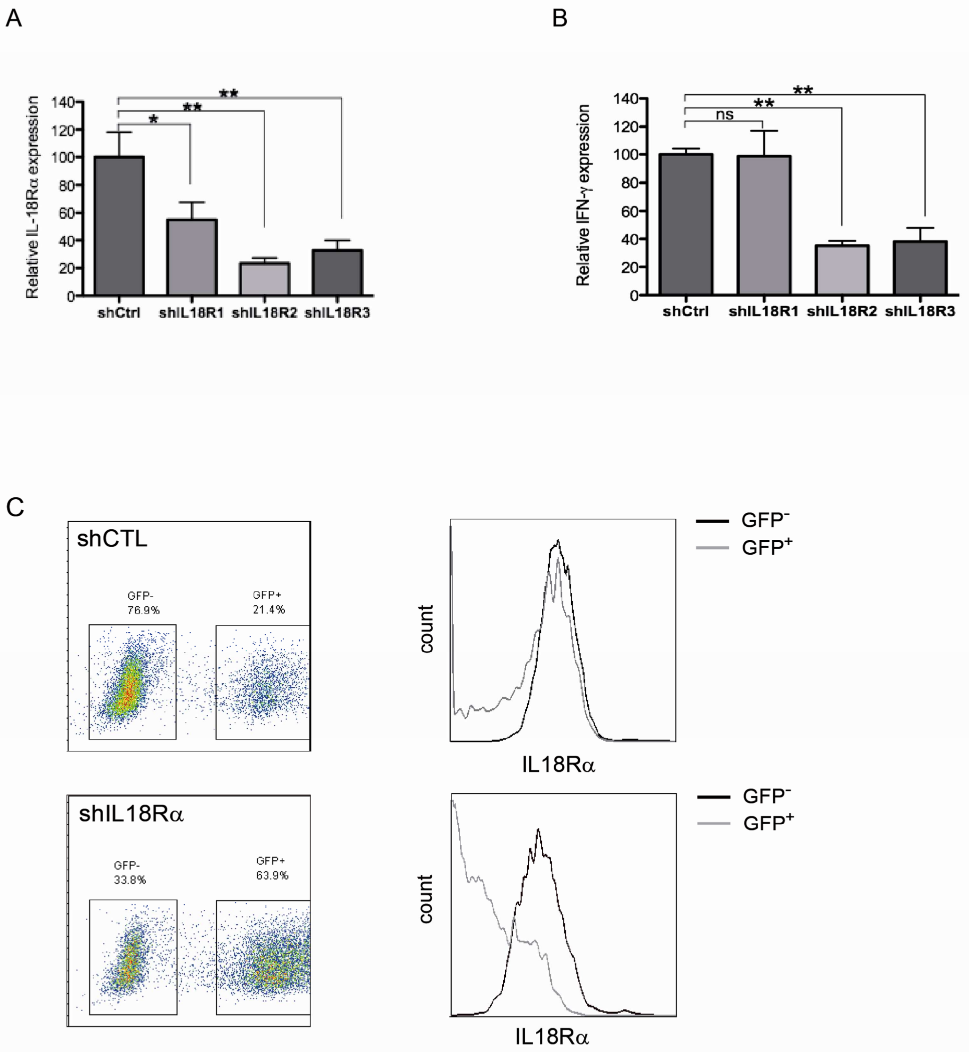

Figure S7: Efficient conditions to decrease human IL18Rα expression by shRNA. Three constructs (shIL18R1, R2, R3) were tested in Peer cells (a human lymphoid cell line from a patient with T-leukemia) that constitutively express IL18R chains. Efficient decrease of expression was observed with all constructs (A), and shIL18R2 and shIL18R3 also affected IFNγ expression (B). The construct shIL18R2 was chosen to transduce primary T-ALL cells. It efficiently decreased IL18Rα cell surface expression in Peer T-ALL cells (C). (Mann and Whitney non-parametric test was used for statistics)
