# Supplementary material for: Interleukin-18 produced by bone marrow-derived stromal cells supports T-cell acute leukaemia progression
Source: EMBO Mol Med. 2014 Apr 28;6(6):821–34. doi: 10.1002/emmm.201303286 (PMC4203358; doi:10.1002/emmm.201303286)
Supplement: Supplementary file 8 — Supplementary Figure S8 [file emmm0006-0821-sd8.pdf]

A

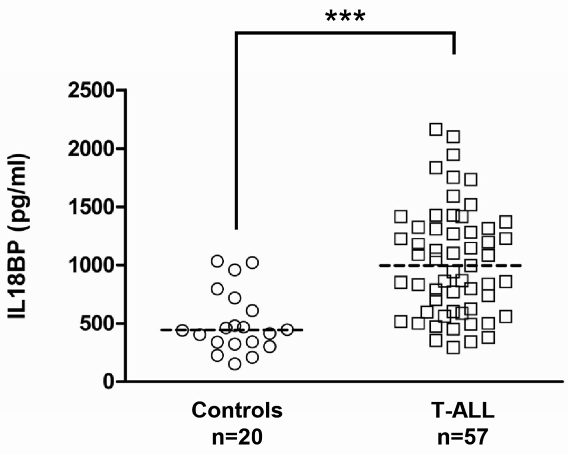

B

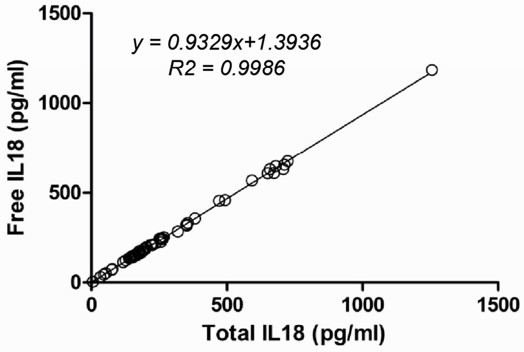

Figure S8: Levels of IL18BP and free IL18 in T-ALL.

Plasma from T-ALL patients (n=57) and controls (n=20) were screened for IL18BP using an ELISA assay. The patients and controls were all children and chosen from the cohort tested for IL18 levels shown in Figure 5C in function of the availability of enough volume of plasma left for every individual. The results show that, as for IL18, IL18BP levels are increased in T-ALL compared to normal (A). Calculation of free IL18 (i.e. not bound to IL18BP) according to previously published works (Novick, Cytokine, 2001) shows a perfect correlation between total and free IL18 levels (B). (Mann and Whitney non-parametric test was used for statistics)
