# Supplementary material for: Interleukin-18 produced by bone marrow-derived stromal cells supports T-cell acute leukaemia progression
Source: EMBO Mol Med. 2014 Apr 28;6(6):821–34. doi: 10.1002/emmm.201303286 (PMC4203358; doi:10.1002/emmm.201303286)
Supplement: Supplementary file 9 — Supplementary Figure S9 [file emmm0006-0821-sd9.pdf]

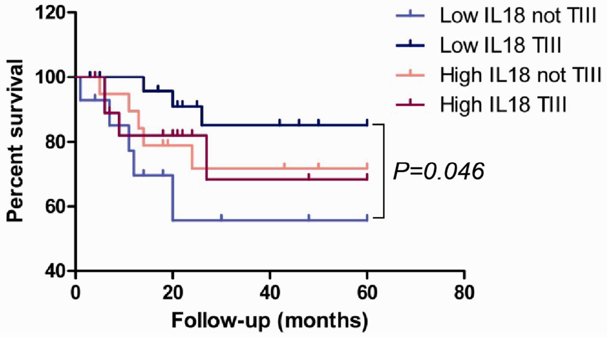

Figure S9. Disease-free survival curves of patients with high or low IL18 plasma levels stratified as EGIL TIII and EGIL not-TIII. The significance was analysed using a Cox-regression model.
