# Supplementary material for: Interleukin-18 produced by bone marrow-derived stromal cells supports T-cell acute leukaemia progression
Source: EMBO Mol Med. 2014 Apr 28;6(6):821–34. doi: 10.1002/emmm.201303286 (PMC4203358; doi:10.1002/emmm.201303286)
Supplement: Supplementary file 10 — Supplementary Table S1 [file emmm0006-0821-sd10.pdf]

| Genes | Forward | Reverse |
| --- | --- | --- |
| GAPDH | GGGAAACTGTGGCGTGAT | GGAGGAGTGGGTGTCGCTGTT |
| β2m | CACAGCCCAAGATAGTTAAGT | CCAGCCCTCCTAGAGC |
| mouse IL18 | CAGGCCTGACATCTTCTGCAA | TCTGACATGGCAGCCATTGT |
| IL18 Rα | TCTTGGACCAAAGCTTAACCA | AAGCAGAGCAGTTGAGCCTTA |
| IL18 Rβ | TTCCGCATCACATAAGCAAG | AAAGACCCTTTCAAAGCCAAA |
| IFNγ | ACTGACTTGAATGTCCAACGCA | ATCTGACTCCTTTTTCGCTTCC |

**Table S1**: Sequences of primers used for real-time quantitative PCR
