# Supplementary material for: Interleukin-18 produced by bone marrow-derived stromal cells supports T-cell acute leukaemia progression
Source: EMBO Mol Med. 2014 Apr 28;6(6):821–34. doi: 10.1002/emmm.201303286 (PMC4203358; doi:10.1002/emmm.201303286)
Supplement: Supplementary file 11 — Supplementary Table S2 [file emmm0006-0821-sd11.pdf]

T-ALL samples	Sex	EGIL maturation stage	White blood cells (G/L)	NOTCH1 mutations	Oncogenic transcripts
M18 R	M	Mature	102	PEST	SIL-TAL1
M22 R	M	Cortical	375	WT	NEG
M30 R	F	Cortical	110	PEST	NEG
M34 R	M	Mature	182	PEST	HOX11ff
M40 R	M	Mature	485	HD	TAL1
M69 R	F	pre-T	238	WT	SIL-TAL1
M78 R	M	Cortical	100	HD	MLL-ENL
M80	F	pro-T	281	PEST	TLX1
M82	F	Mature	194	PEST	LMO2
M86 R	M	Mature	5	PEST	NEG
M87	M	pre-T	168	nd	SIL-TAL1/ LMO2
M88	M	Mature	152	nd	TLX3
M89	F	Mature	2	nd	TAL1
M97 R	M	Cortical	154	nd	nd
M100	M	Mature	244	HD	TLX3
M105 NR	M	Cortical	129	HD	TAL1/ LMO2
M106 R	F	Mature	743	WT	SIL-TAL1/ LMO2
M108 R	M	Cortical	707	HD+PEST	c-Myb
M109 NR	M	pre-T	115	WT	TAL1/ LMO2 / PTEN
M110 R	M	Cortical	290	PEST	NEG
M112 NR	F	Mature	109	WT	TLX3
M114 NR	M	Cortical	362	PEST	NEG
M118 R	M	Cortical	460	WT	TAL1/ LMO2 / PTEN
M120 R	F	pre-T	439	WT	SIL-TAL1
M121 NR	F	pre-T	108	WT	LMO2
M129 NR	F	Cortical	7	PEST	NEG

Table S2: Summary of the characteristics of the human T-ALL samples used for the experiments described in Figures 1, 2, 3, 4A and 5. Each T-ALL sample was coded (M + number) as mentioned in previous studies (Armstrong, Blood, 2009; Gerby, Leukemia, 2011). R and NR mean respectively MEKi-responsive and non-responsive. Samples without R and NR are samples which did not grow in culture.
