# Supplementary figures and images for: Interleukin-18 produced by bone marrow-derived stromal cells supports T-cell acute leukaemia progression
Source: EMBO Mol Med. 2014 Apr 28;6(6):821–34. doi: 10.1002/emmm.201303286 (PMC4203358; doi:10.1002/emmm.201303286)

**Figure 2A**  
(left panel)

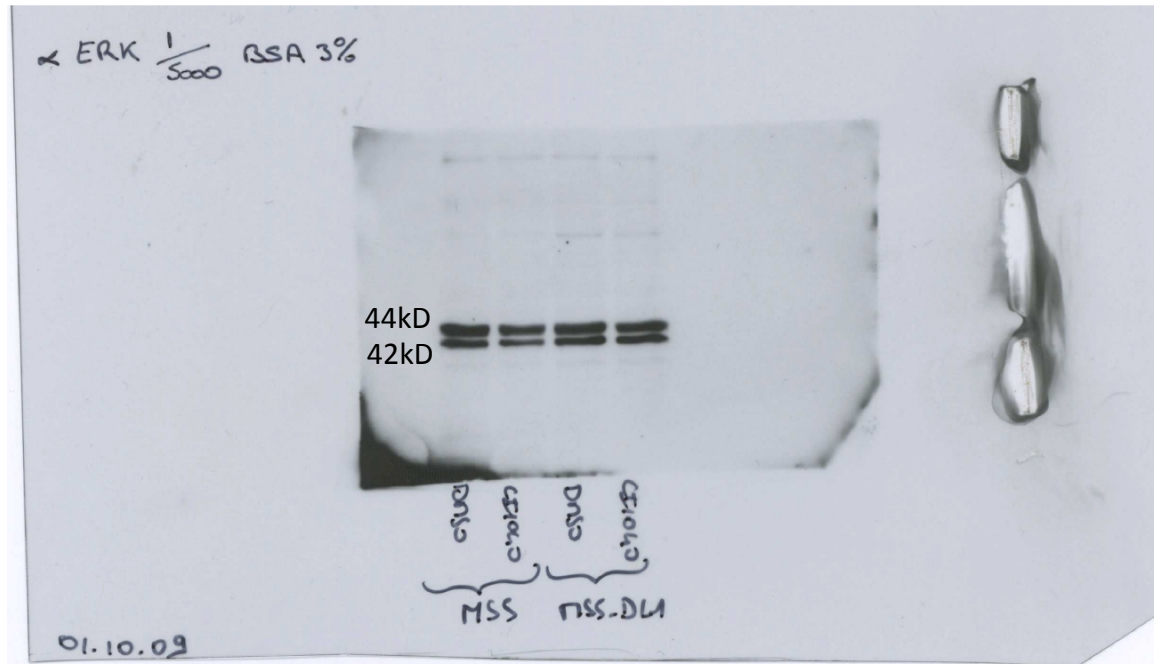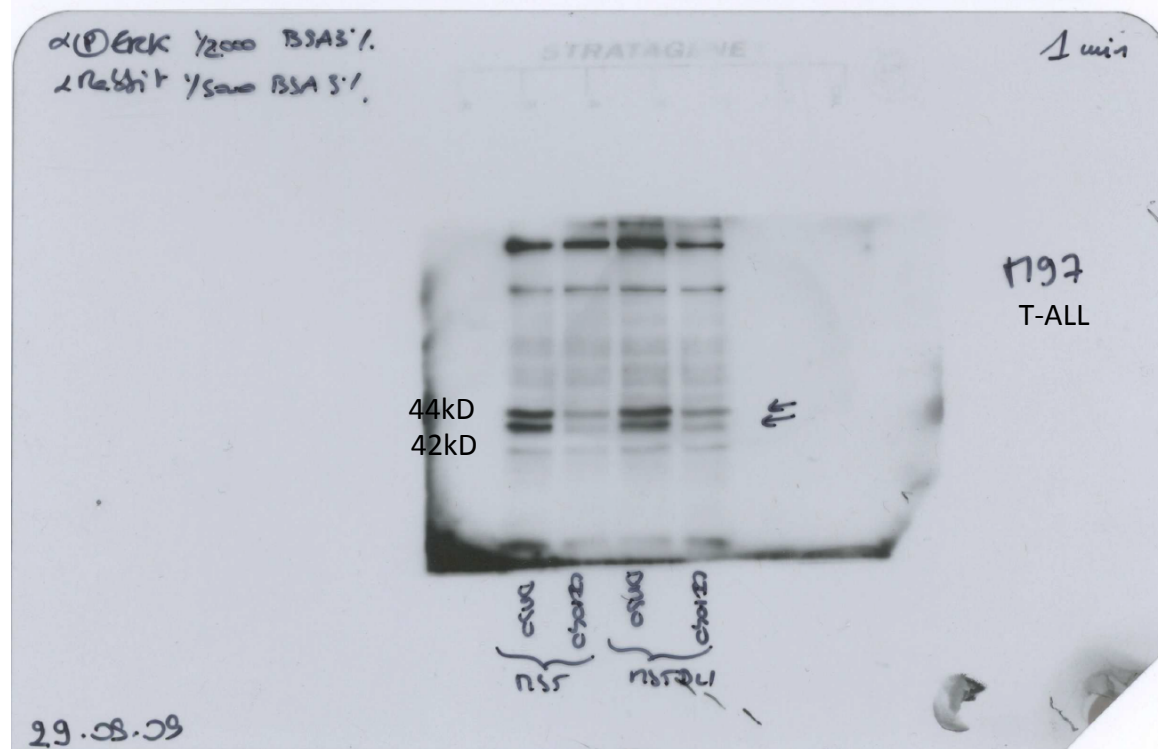

**Figure 2A**  
(right panel)

Western blot on MS5 stromal cells

Total ERK

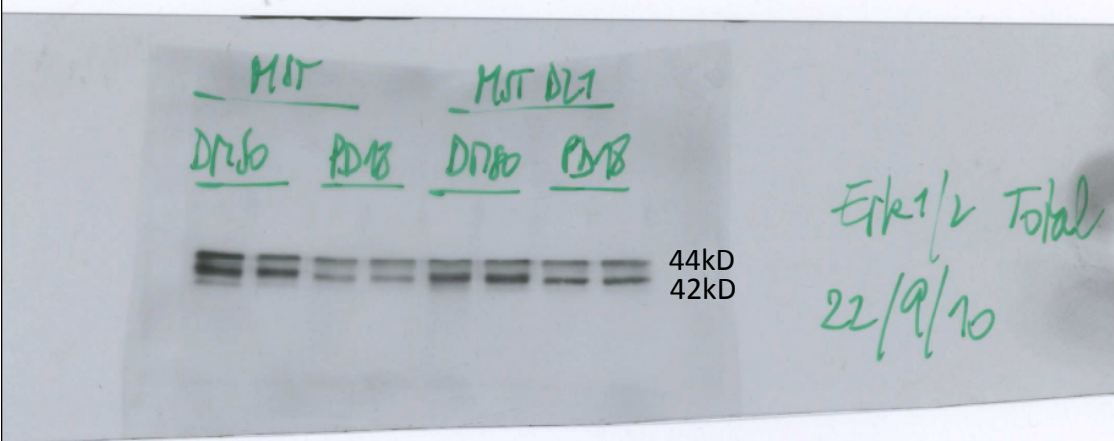

Phospho-ERK

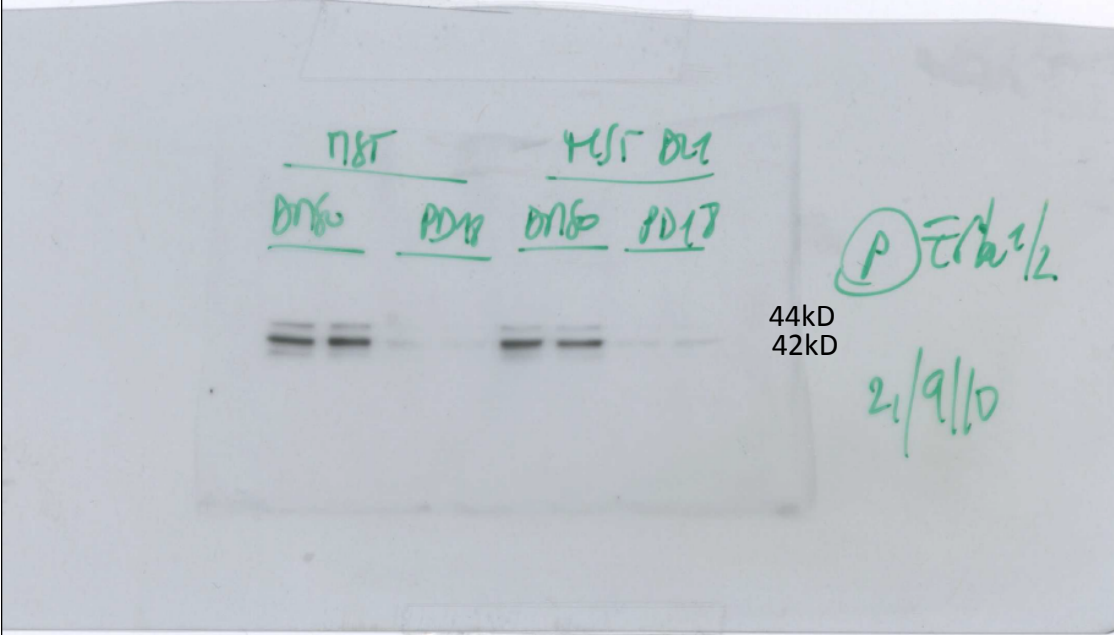

Supplement: Supplementary file 13 — Source Data for Figure 2A [file emmm0006-0821-sd13.pdf]
