# Supplementary material for: Interleukin-18 produced by bone marrow-derived stromal cells supports T-cell acute leukaemia progression
Source: EMBO Mol Med. 2014 Apr 28;6(6):821–34. doi: 10.1002/emmm.201303286 (PMC4203358; doi:10.1002/emmm.201303286)

Figure 2B (insert)

Western blot from MS5 stromal cells

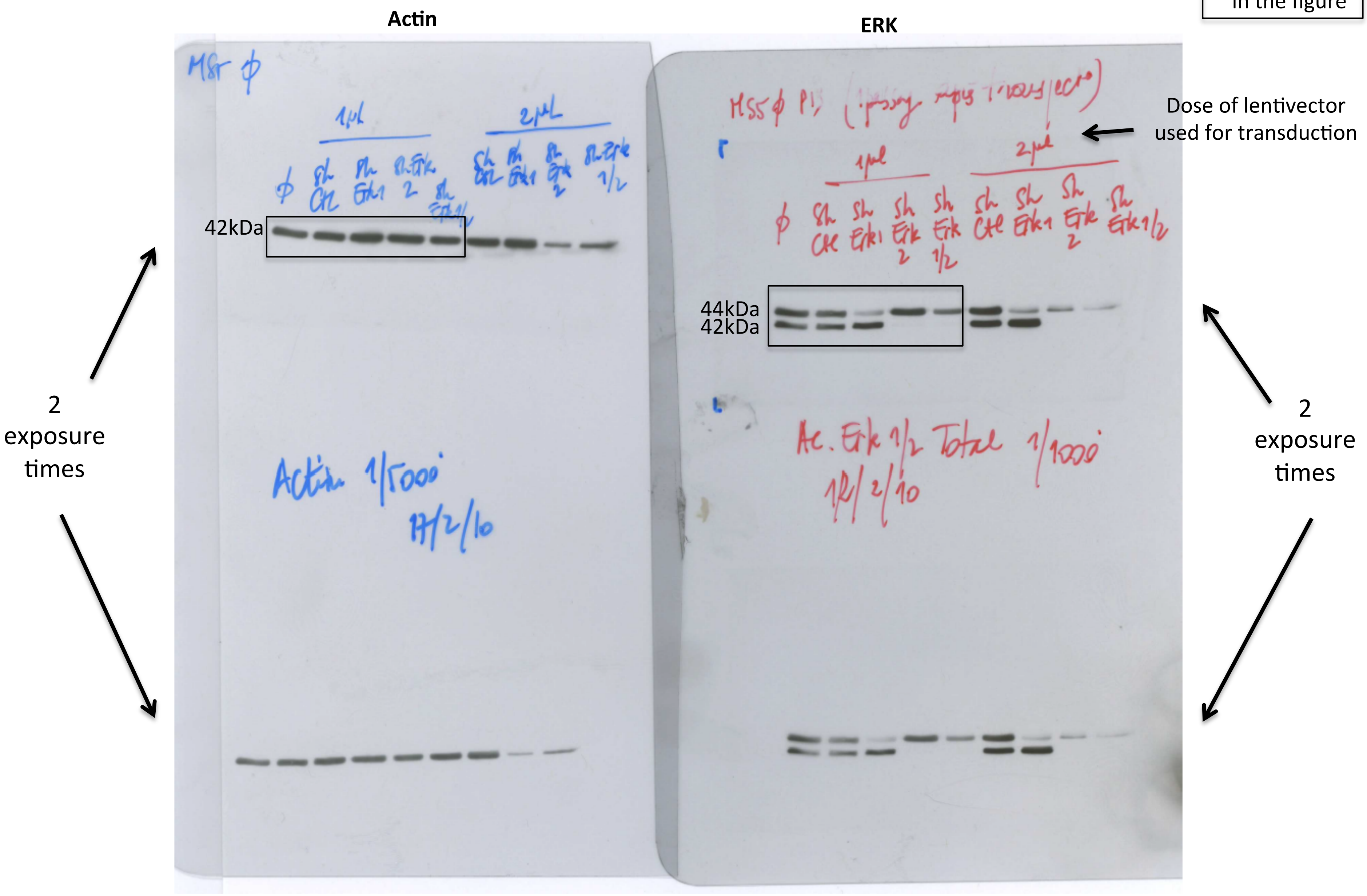

Supplement: Supplementary file 14 — Source Data for Figure 2B [file emmm0006-0821-sd14.pdf]
